# Two‐in‐one method: Novel pancreaticojejunostomy technique for the bifid pancreas

**DOI:** 10.1002/ags3.12302

**Published:** 2019-12-23

**Authors:** Jun Ishida, Hirochika Toyama, Daisuke Tsugawa, Masahiro Kido, Takumi Fukumoto

**Affiliations:** ^1^ Division of Hepato‐Biliary‐Pancreatic Surgery Department of Surgery Kobe University Graduate School of Medicine Kobe Japan

**Keywords:** bifid pancreas, pancreaticoduodenectomy, pancreaticojejunostomy

## Abstract

The bifid pancreas is a rare anatomical variation of the pancreatic duct in which double main pancreatic ducts in the body and tail of the pancreas join at the pancreas head and drain through the major papilla. When pancreaticoduodenectomies are carried out on bifid pancreases, close attention must be paid to the reconstruction because of the possibility that there may be two pancreatic ducts that need to be reconstructed. We present a case of pancreaticoduodenectomy for the bifid pancreas and a novel technique named the ‘two‐in‐one’ method for double pancreatic duct to jejunum anastomosis. Using the two‐in‐one method, we anastomosed one jejunal hole to a double pancreatic duct. Pancreatic texture was normal and postoperative volumes of pancreatic juice from the two external pancreatic duct stents were 250 mL and 100 mL/day, respectively. Postoperative recovery went well although the patient needed a slightly longer hospital stay as a result of surgical site infection. This novel anastomotic technique was as simple to carry out as a normal pancreaticojejunostomy and may be useful for reconstruction of the bifid pancreas.

## Graphical abstract

We present a novel technique named the ‘two‐in‐one’ method for double pancreatic duct to jejunum anastomosis for the bifid pancreas. Using the two‐in‐one method, we anastomosed one jejunal hole to a double pancreatic duct. This novel anastomotic technique was as simple to carry out as a normal pancreaticojejunostomy and may be useful for reconstruction of the bifid pancreas.

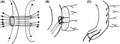

## 1 INTRODUCTION

The bifid pancreas is a rare anatomical variation of the pancreatic duct in which double main pancreatic ducts in the body and tail of the pancreas join at the pancreas head and drain through the major papilla.^1^, ^2^, ^3^, ^4^, ^5^, ^6^, ^7^, ^8^, ^9^ Most cases are asymptomatic and are detected incidentally by magnetic resonance cholangiopancreatography (MRCP) or endoscopic retrograde cholangiopancreatography (ERCP). However, when pancreaticoduodenectomies (PD) are carried out on patients with bifid pancreases, close attention should be paid to the method of pancreaticojejunostomy. Herein, we present a case of PD in a patient with a bifid pancreas which was diagnosed during surgery, and a new technique of double pancreatic duct to jejunum anastomosis, which we call the ‘two‐in‐one’ method.

## 2 MATERIALS AND METHODS

### 2.1 Case presentation

**Figure 1:**
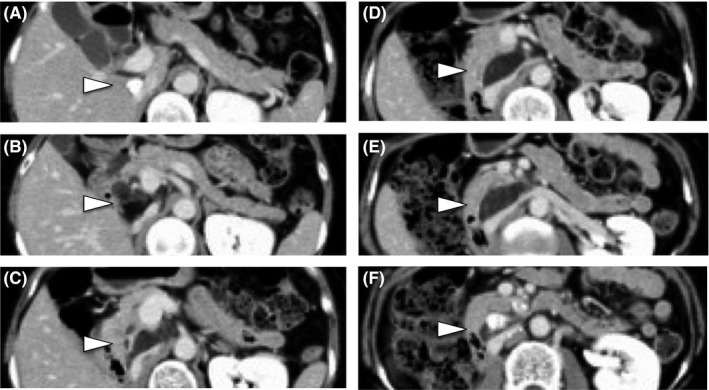
Preoperative computed tomography images. (A‐F) There is a low‐density mass including calcification around the inferior vena cava behind the liver and the head of the pancreas. Arrowheads in A‐F in figure 1 are pointing to a low density mass including calcification

A 59‐year‐old woman made regular visits to our hospital for follow up after resection of a retroperitoneal liposarcoma. Follow‐up computed tomography (CT) showed a low‐density mass, including calcification, around the inferior vena cava behind the liver and the head of the pancreas (Figure 1). Blood test showed no evidence of elevated serum tumor markers. We diagnosed her with a recurrence of the liposarcoma and planned tumor resection with a PD if needed. The patient did not undergo MRCP because she did not have a primary pancreatic tumor.

**Figure 2:**
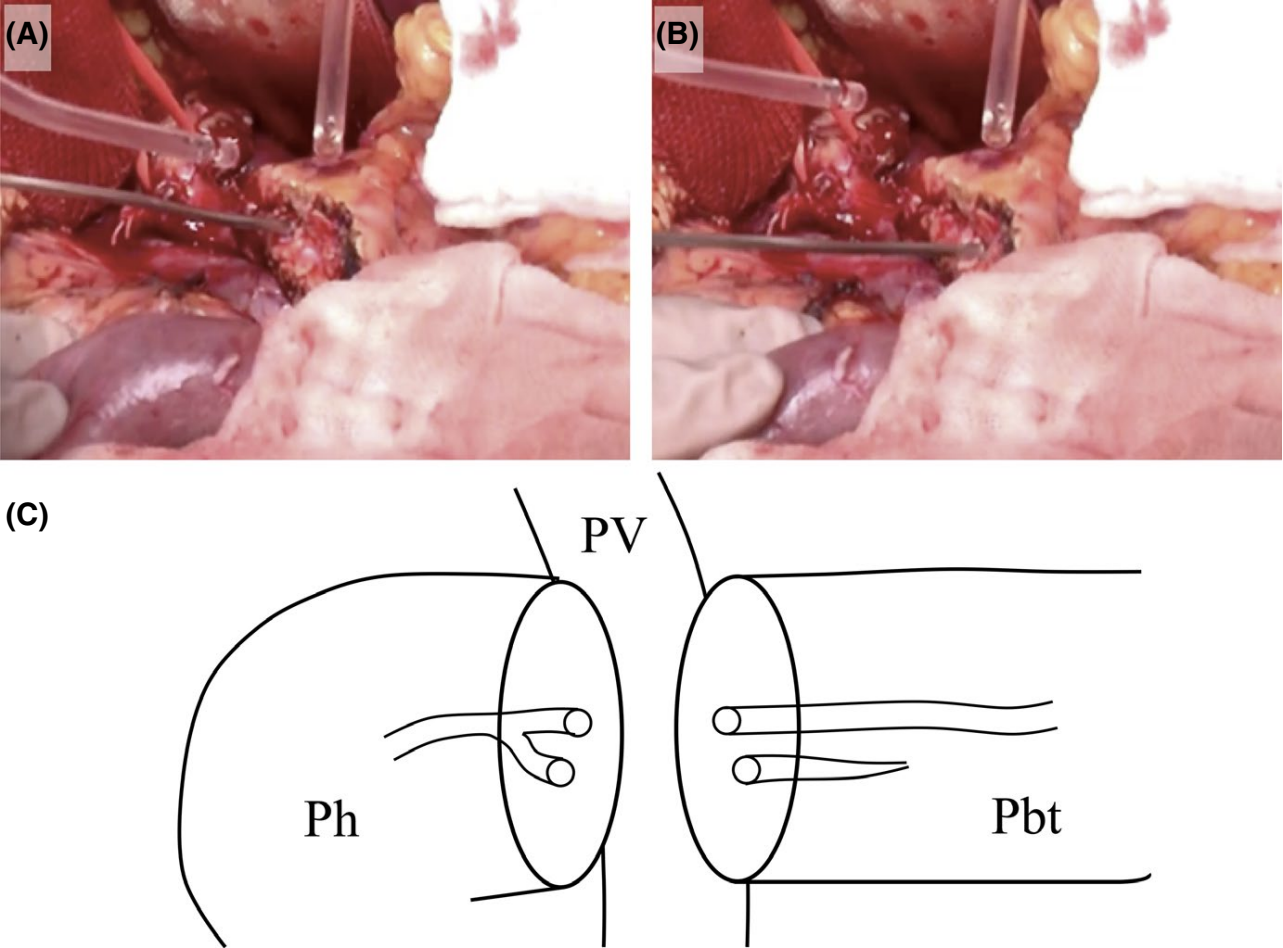
Double pancreatic duct orifices were found on the cut surface of the residual pancreas. A, Blunt tip probe could be inserted deep into the cranial duct. B, Blunt tip probe could be inserted 3 cm into the caudal duct. C, Schema after transection of the pancreas. Double pancreatic ducts joined near the cut surface of the pancreas head. Distance between the two ducts on the cut surface was 3 mm. Pbt, body and tail of the pancreas; Ph, head of the pancreas; PV, portal vein

**Figure 3:**
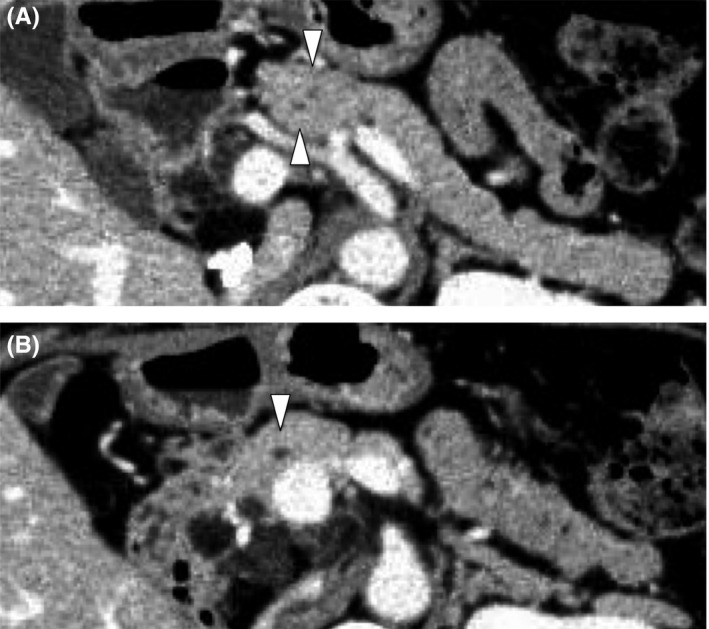
Preoperative multi‐detector computed tomography shows a bifurcated double main pancreatic duct. A, There were two pancreatic ducts in the pancreas body. B, Two ducts joined at the level of the portal vein. Arrowheads in part A shows double main pancreatic duct. Arrowhead in part B shows the main pancreatic duct on the left side of the confluence of double pancreatic duct

Because the operative findings showed strong adhesion between the recurrent tumor and the pancreas, we decided to carry out PD. We transected the pancreas upon the portal vein. Following transection, subsequent operative exploration of the cut surface of the residual pancreas identified a double pancreatic duct orifice (approximately 3 and 2 mm in diameter, respectively). A bifid pancreatic duct was subsequently confirmed by intraoperative probing using blunt‐tipped probes, which could be inserted deep into the cranial duct and 3 cm into the caudal duct of the residual pancreas (Figure 2A,B). The double pancreatic ducts joined near the cut surface of the resected head of the pancreas. Distance between the two ducts on the cut surface was 3 mm (Figure 2C). Preoperative multi‐detector computed tomography (MDCT) showed a bifurcated double main pancreatic duct, which we had not noticed before the operation (Figure 3).

### 2.2 Surgical techniques of the two‐in‐one method

**Figure 4:**
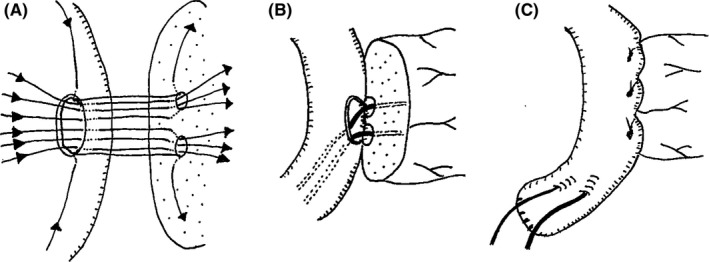
Two‐in‐one method for double pancreatic duct to jejunum anastomosis with transpancreatic jejunal sutures (modified Blumgart mattress suture technique). (A,B) We carried out posterior sutures of the double pancreatic duct‐to‐jejunum anastomosis using four threads for each duct, followed by a 4‐Fr external pancreatic duct stent insertion into each duct. C, After the anterior sutures, using three threads for each duct, we sutured through the seromuscular layer of the jejunal anterior wall using a modified Blumgart mattress suture technique tied at the ventral wall of the jejunum to completely cover the pancreatic stump with the jejunal serosa

We carried out a double pancreatic duct to jejunum anastomosis with transpancreatic jejunal sutures (a modification of the Blumgart mattress suture technique).^10^ We placed three sutures through the transpancreatic and the seromuscular layer of the jejunal posterior wall on the long axis using 3‐0 ASFLEX (Kono Seisakusho, Ichikawa, Japan) sutures, followed by a double duct to mucosa anastomosis using 5‐0 PDS (Ethicon, Tokyo, Japan) sutures. We placed a hole 5 mm in diameter in the jejunum and sutured through the cranial edge and caudal edge of the jejunal hole. Posterior sutures of the double pancreatic duct to jejunum anastomosis were done using four threads for each duct, followed by a 4‐Fr external pancreatic duct stent insertion into each duct. After the anterior sutures, using three threads for each duct, we sutured through the seromuscular layer of the jejunal anterior wall using sutures used for posterior sutures of a modified Blumgart mattress suture, and tied at the ventral wall of the jejunum to completely cover the pancreatic stump with jejunal serosa (Figure 4).

## 3 RESULTS

After the operation, volumes of pancreatic juice from the two external pancreatic duct stents were 250 and 100 mL/day, respectively. Although amylase level in the drainage fluid was high (6926 U/L) on postoperative day (POD) 1, it decreased to 526 U/L on POD3. The drainage tube was replaced on POD 15 because the drainage fluid showed signs of infection and was removed on POD 34. Two external pancreatic duct stents were clamped on POD 29 and 31, respectively. The patient was discharged on POD 39. Both external pancreatic duct stents were removed in an outpatient clinic.

## 4 DISCUSSION

The bifid pancreas is a rare anatomical variation which is reported to be present in the population at a rate of 0.9%‐2.7%.^11^, ^12^ Halpert et al first reported a patient with bifid pancreas, diagnosed by ERCP, in 1990.^9^ Because most patients are asymptomatic, it is rarely diagnosed without MRCP or ERCP if the duplicated pancreatic ducts are not dilated. In this case, we diagnosed the bifid pancreas by reviewing the MDCT after transection of the pancreas. We carried out a double pancreatic duct to jejunum anastomosis, which we named the ‘two‐in‐one’ method. Postoperative recovery went well although she needed a slightly longer hospital stay as a result of surgical site infection.

In the two‐in‐one method, two pancreatic ducts are anastomosed to one jejunal hole. We carried out this method because of the short distance between the two ducts on the cut surface of the pancreas. In hepaticojejunostomy, multiple bile ducts can be anastomosed to one jejunal hole by plasty of neighboring ducts. In addition, the Glissonean sheath, including multiple bile ducts, could be treated as a single duct by regarding the septa as a thick wall of the duct.^13^ However, in pancreaticojejunostomy for the bifid pancreas, we cannot carry out plasty because the two ducts are not in contact with each other. Up until now, there have been only a few reports of pancreaticojejunostomy for double pancreatic ducts. There are four reported cases of double duct to mucosa pancreaticojejunostomy for the bifid pancreatic duct following PD.^1^, ^2^, ^3^ Shim et al also reported a case of PD for the bifid pancreas in which they sutured and ligated the smaller duct and carried out a routine pancreaticojejunostomy.^4^ Because there is no established method for pancreaticojejunostomy for double pancreatic ducts, surgeons should carry out the reconstruction method deemed most appropriate for each patient. When the distance between the two ducts on the cut surface of the pancreas is short, because we cannot place two jejunal holes too close together, the conventional anastomosis technique of two pancreatic ducts to two jejunal holes may be inappropriate. Suturing the tiny jejunal wall between the two holes may also be difficult. We consider that the two‐in‐one method may be preferable when the distance between the two ducts is 5 mm or shorter.

**Figure 5:**
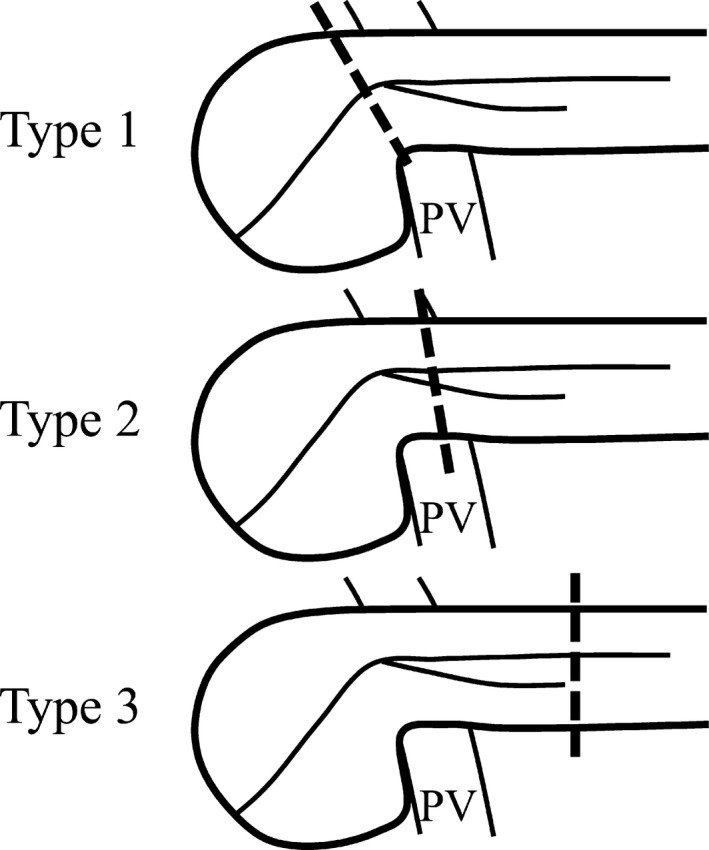
Three types of pancreatic transections and reconstructions for the bifid pancreas based on the location. Type 1: Pancreatic transection on the proximal side of the confluence of the duplicated pancreatic ducts enables surgeons to carry out a normal pancreaticojejunostomy. Type 2: Pancreatic transection on the distal side of the confluence of the duplicated pancreatic ducts enables surgeons to carry out a pancreaticojejunostomy for the double pancreatic ducts. Type 3: Pancreatic transection on the distal side of the terminal of the shorter pancreatic duct enables surgeons to carry out a normal pancreaticojejunostomy. PV, portal vein

When we plan to carry out PD for patients with bifid pancreases, we should consider three types of pancreatic transection and reconstruction based on the location (Figure 5). Type 1 is a pancreatic transection on the proximal side of the confluence of the duplicated pancreatic ducts. This method enables surgeons to carry out normal pancreaticojejunostomy. Ohkubo et al reported a case of PD for the bifid pancreas in which they carried out a single pancreatic duct pancreaticojejunostomy by transecting the pancreas on this level.^5^ Type 2 is a pancreatic transection on the distal side of the confluence of the duplicated pancreatic ducts. For reconstruction, surgeons should choose from two methods: ligation of the smaller pancreatic duct or pancreaticojejunostomy for the double pancreatic ducts. Type 3 is a pancreatic transection on the distal side of the terminal of the shorter pancreatic duct. This type of reconstruction should be considered when we cut the pancreas on the left side and can achieve a negative margin for the tumor. In type 3, a normal pancreaticojejunostomy can be done, but decreased pancreatic function as a result of the small residual pancreas should be taken into account. For the bifid pancreas, we should cut the pancreas differently in each patient, taking into consideration the location of the confluence of the double ducts, the length of the shorter duct, and tumor location.

In the present case, we cut the pancreas on the level of type 2. We did not ligate one of the double ducts because the patient had a normal pancreatic parenchyma and pancreatic juice flowed from both pancreatic ducts. We considered an additional pancreatic resection for type 3 reconstruction but opted to preserve pancreatic function instead. In this patient, ligation of the shorter duct would possibly have resulted in postoperative pancreatitis because 100 mL/day of pancreatic juice flowed from the external pancreatic duct stents inserted into the shorter duct.

In conclusion, clinicians should always be aware of the possibility of a bifid pancreas. If a bifid pancreas is encountered during an operation, an appropriate reconstruction method should be chosen based on the length of the shorter duct, pancreatic function, and the tumor margin. Our unique two‐in‐one method may be a useful reconstruction method for the bifid pancreas.

## DISCLOSURE

Conflicts of Interest: Authors declare no conflicts of interest for this article.

Author Contribution: Study conception and design: Ishida, Toyama, Tsugawa, Kido, Fukumoto. Drafting of the manuscript: Ishida, Toyama. Critical revision: Fukumoto. Final approval for publication: Ishida, Toyama, Tsugawa, Kido, Fukumoto.
